# Remote diffusion-weighted imaging lesions and blood pressure variability in primary intracerebral hemorrhage

**DOI:** 10.3389/fneur.2022.950056

**Published:** 2022-09-20

**Authors:** Xuhua Xu, Shuangshuang Peng, Yanli Zhou, Jiawen Li, Lusha Tong, Feng Gao

**Affiliations:** ^1^Department of Neurology, The Second Affiliated Hospital, Zhejiang University School of Medicine, Hangzhou, China; ^2^Department of Neurology, The Fourth Affiliated Hospital, Zhejiang University School of Medicine, Yiwu, China; ^3^Department of Neurology, Taizhou First People's Hospital, Taizhou, China

**Keywords:** intracerebral hemorrhage, remote DWI lesions, blood pressure, standard deviation, coefficient of variation, successive variation

## Abstract

**Objective:**

The aim of this study was to examine the association between remote diffusion-weighted imaging lesions (R-DWILs) and blood pressure variability (BPV) in patients with primary intracerebral hemorrhage (ICH).

**Methods:**

We conducted a retrospective review of a consecutive cohort of 375 patients with primary ICH within 24 h onset. R-DWILs were defined as hyperintensity lesions in DWI remote from the hematoma. Blood pressure recordings were extracted up to 24 h post-admission. BPV was measured using SD, coefficient of variation (CV), and successive variation (SV).

**Results:**

Remote DWI lesions were detected in 65 (17.3%) primary ICH patients. In multivariable logistic regression analysis, parameters of BPV were independently associated with R-DWILs, and the results remained consistent after being adjusted with mean SBP. SD, CV, and SV values in the highest quintile, showed 3- to 8-fold increased risk of R-DWILs, compared with the lowest quintile. ΔSBP demonstrated a significant difference in 2 different predictive models. Max SBP only dictated a significant difference in model 1. Mean SBP, admission SBP, and min SBP, failed to present an association with R-DWILs in model 1 or model 2.

**Conclusion:**

Our results provided additional evidence that BPV is associated with the development of R-DWILs in primary ICH.

## Introduction

Remote lesions identified using diffusion-weighted imaging (DWI) in primary intracerebral hemorrhage patients have been reported with an incidence of 11–41% (1). These DWI lesions (R-DWILs) are described as small, cortical, or subcortical, and are considered to be associated with worse outcomes (2). However, the mechanism and etiology of these lesions remain unclear.

It has been argued that aggressive blood pressure (BP) lowering in acute ICH may increase the possibility of cerebral hypoperfusion, and thus induce cerebral ischemia (3–5). Nevertheless, the causal relationship of BP lowering the risk of R-DWILs in acute ICH has not been clearly demonstrated yet. While several studies have demonstrated a positive correlation between BP lowering and R-DWILs (6–8), others have failed to find the relationship between them (2, 9, 10). However, these analyses were based on only limited parameters and few time point measurements of blood pressure, or just focused on the parameters of maximum BP, minimum BP, and maximum BP minus minimum BP.

There is increasing evidence supporting that not only the absolute value of BP values but also their variations over time contributing to cerebrovascular events, as well as the outcome (11, 12). Therefore, the aim of this study was to clarify the association of BP variability (BPV) during the first 24 h after primary ICH and the remote DWI lesions in a cohort having continuous BP measurements.

## Methods

### Study population

We reviewed our prospectively collected data from a consecutive enrolled patient cohort with acute primary ICH who were admitted to the department of neurology at the Second Affiliated Hospital of Zhejiang University between November 2016 and October 2019. Patients were included in this study if they had acute primary ICH within 24 h of symptom onset, and magnetic resonance imaging (MRI), including DWI, apparent diffusion coefficient (ADC), and fluid-attenuated inversion recovery (FLAIR) sequences. Patients with ICH due to aneurysms, vascular malformations, moyamoya disease, cavernous hemangioma, head trauma, cerebral venous thrombosis, hemorrhagic transformation after ischemic infarction, or neoplasm were excluded. Patients were also excluded if they had inadequate BP data. The study protocol was approved by the institutional Human Research Ethics Committee of the Second Affiliated Hospital of Zhejiang University.

### Baseline data

Demographic data (age, gender, time from symptom onset to MRI), medical history [hypertension, diabetes mellitus, atrial fibrillation (AF), coronary heart disease (CHD) previous ischemic stroke or transient ischemic attack (IS/TIA), previous ICH, smoking, alcohol], laboratory results [hemoglobin, platelet, white blood cell (WBC), total cholesterol (TC), low-density lipoprotein cholesterol (LDL-C), fasting blood glucose (FBG), C-reactive protein (CRP)], as well as clinical assessments [National Institutes of Health Stroke Scale (NIHSS) scores on admission] were collected in all patients. Hematoma volume was calculated using the ABC/2 method based on initial computed tomography (CT) scan (13), and hematoma locations were categorized as lobar, deep, or mixed (presence of lobar and deep hematoma simultaneously).

### Blood pressure (BP) measurements and parameters

All patients underwent on admission a non-invasive BP monitoring (Mindray IMEC8, Shenzhen, China) over the first 24 h after symptoms onset. Standard arm cuffs suitable for arm circumferences ranging from 25 to 35 cm were supplied. The supine BP was measured in the non-paretic arm using an oscillometric method, which is clinically proven to produce fast and reliable results. Systolic BP (SBP) and diastolic BP (DBP) readings were obtained every 2 h in the first 24 h. The parameters of BP variability were derived manually, including SD, coefficient of variation (CV, defined as SD/mean ×100), and successive variation (SV). SV is the square root of the average difference in BP between successive measurements. We also measured admission, average (mean), maximum (max), minimum (min), and difference between max and min (Δ) of BP. Treatment of BP was performed according to guideline recommendations and the physician' discretion. We screened for patients who were prescribed antihypertensive medication in the first 24 h, that was classified as a continuous infusion, intermittent infusion, and oral medication.

### Magnetic resonance image (MRI) acquisition

MRI was performed on 1.5-Telsa (Sonata, Siemens, German) with a standardized protocol consisting of axial T1-weighted, T2-weighted, T2 FLAIR, DWI, and apparent diffusion coefficient (ADC) sequences. Axial DWI sequences were acquired based on following parameters: repetition time [TR] 3,100 ms, echo time [TE] 84 ms, *b* = 0/1000s/ mm^2^, 6-mm slice thickness, 0.5-mm gap, FOV 230 mm, base resolution 176, phase resolution 100%, voxel size, 1.3 ×1.3 ×6 mm.

Remote DWI lesions were defined as hyperintensity lesions in DWI and <20 mm in diameter, with a corresponding hypo-intensity in the ADC map (14). Those DWI lesions which are close to the hematoma (<20 mm) were excluded. Two neurologists of the stroke specialty (LT and FG) read the data of R-DWILs independently and reached a consensus (Kappa = 0.94).

White matter hyperintensity (WMH) was identified as hyperintensity on FLAIR sequences (15). Periventricular white matter hyperintensity (PWMH) and deep white matter hyperintensity (DWMH) were assessed, respectively, according to the Fazekas scale with scores grading from 0 to 3. A total score >2 was defined as high-grade WMH in the present study.

### Outcomes evaluation

We collected standardized functional outcomes [modified Rankin Scale (mRS)] at 3 months by telephone or in-person interviews. An unfavorable outcome was defined as mRS of 4–6 at 3 months. All outcome data were obtained by examiners certified in the mRS. The examiners were blinded to each patient's clinical course and radiographic imaging.

### Statistical analysis

In our study, categorical variables were presented as percentages, and continuous variables were presented as mean standard deviation if normally distributed or median (interquartile range) if not normally distributed, which were checked by the Shapiro–Wilk test. We compared the demographic, clinical characteristics, and BPV parameters of patients with and without R-DWILs using the χ^2^ test and Fisher's exact test for categorical variables, and the independent-sample *t* test or the Mann–Whitney *U* test for continuous variables, as appropriate. The proportion of patients with R-DWILs was then compared according to quintiles of each of the BPV parameters (Q1 means lowest quintile group and Q5 means highest quintile group). Finally, binary logistic regression analysis was performed to assess the independent association of each BP parameter, as quintiles, with the presence of R-DWILs. Model 1 involved variables with *p* < 0.10 in the univariate analysis as well as those which are known to have a definite association with the R-DWILs in clinical practice. Because BPV increases proportionally to the increased BP level, model 2 was adjusted using mean BP besides model 1 variables. All covariates were proved to be independent using the likelihood ratio test. To avoid the risk of multicollinearity caused by including highly correlated variables in the same model, we replaced each variability parameter one by one instead of including all the BP variability parameters in one model. Regarding that binary regression might create artificially significant values, Spearman's correlation analysis was performed to explore associations between BPV parameters, as continuous variables, and R-DWILs numbers. Then, multiple linear regression analysis was used with the covariates mentioned above. All the assumptions for linear regressions were met. We also undertook an exploratory analysis to compare BP variability in patients with mRS scores of 0–3 vs. 4–6. A *p*-value <0.05 was considered to be statistically significant. All statistical analyses were carried out using SPSS Version 24.0 (IBM Corp., Armonk, NY, USA).

## Results

**Figure 1:**
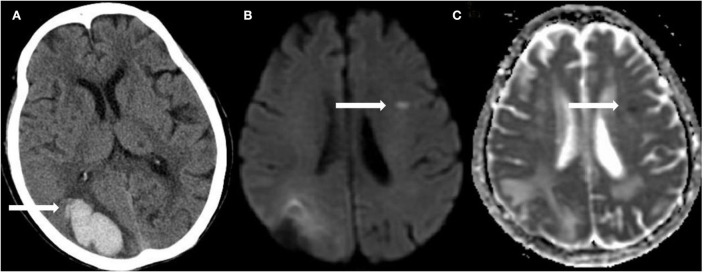
A representative example of R-DWILs. In a 92-year-old female with right occipital lobe hemorrhage **(A)**, diffusion-weighted imaging (DWI) shows a small remote ischemic lesion on the left frontal lobe **(B)**, with corresponding low signal intensity in the apparent diffusion coefficient (ADC) map **(C)**.

Of the 976 cases enrolled within the timeframe of analysis, a total of 515 were included in the final analysis (Supplementary Figure S1 presents the inclusion and exclusion flow chart). Table 1 shows the overall characteristics of the cohort as well as characteristics stratified by the presence or absence of R-DWILs (Figure 1 for a representative case example). Overall, 17.3% of patients had 1 or more DWI lesions (median 1, range 1–8). Their median diameter was 7 mm (range 2–18 mm). At baseline, patients with DWI lesions were older, and more likely to have higher FBG and higher grade WMH (Table 1). The time interval from ICH onset to finishing MRI was comparable between patients with [median 6, IQR (interquartile range) 3] and without R-DWILs (median 5, IQR 3).

**Table 1:** Comparison of baseline characteristics between patients with and without R-DWILs.

	**R-DWILs**			
	**All**	**No**	**Yes**	***P*-value**
*N* (%)	375 (100)	310 (82.7)	65 (17.3)	
Age, year, mean (SD)	61.07	60.48	63.88	0.071
	(13.81)	(13.65)	(14.35)	
Male, *n* (%)	245 (65.3)	203 (65.5)	42 (64.6)	0.894
Hypertension, *n* (%)	284 (75.7)	231 (74.5)	53 (81.5)	0.230
Diabetes mellitus, *n* (%)	53 (14.1)	40 (12.9)	13 (20.0)	0.135
Previous ICH, *n* (%)	30 (8.0)	28 (9.0)	2 (3.1)	0.108
Previous IS/TIA, *n* (%)	33 (8.8)	24 (7.7)	9 (13.8)	0.114
AF, *n* (%)	10 (2.7)	7 (2.3)	3 (4.6)	0.387
CHD, *n* (%)	8 (2.1)	7 (2.3)	1 (1.5)	1.000
Smoking, *n* (%)	120 (32.0)	101 (32.6)	19 (29.2)	0.599
Alcohol, *n* (%)	119 (31.9)	100 (32.4)	19 (29.7)	0.676
Time to MRI, day, median (IQR)	5 (3)	5 (3)	6 (3)	0.378
Initial NIHSS, median (IQR)	5 (8)	5 (8)	5 (9)	0.966
WBC, ×109/l, median (IQR)	8.0 (3.4)	8.0 (3.4)	7.7 (4.0)	0.810
Hemoglobin, g/l, median (IQR)	138 (21)	138 (21)	136 (21)	0.555
Platelet, ×109/l, median (IQR)	194 (79)	194 (78)	194 (98)	0.401
TC, mmol/l, median (IQR)	4.7 (1.3)	4.6 (1.3)	4.8 (1.3)	0.728
LDL-C, mmol/l, median (IQR)	2.4 (1.1)	2.4 (1.1)	2.6 (0.9)	0.230
FBG, mmol/l, median (IQR)	5.9 (1.7)	5.8 (1.7)	6.3 (1.6)	*0.004*
CRP, mg/l, median (IQR)	4.0 (7.4)	3.9 (7.1)	6.0 (9.4)	0.103
Hematoma Volume, ml, median (IQR)	9.0 (13.9)	8.8 (13.0)	10.6 (20.8)	0.411
Hematoma Location, *n* (%)				0.372
Lobar	85 (22.7)	66 (21.3)	19 (29.2)	
Deep	279 (74.4)	235 (75.8)	44 (67.7)	
Mixed	11 (2.9)	9 (2.9)	2 (3.1)	
High-grade WMH, *n* (%)	172 (47.1)	122 (40.4)	50 (79.4)	*<0.001*
Antihypertensive Medication, *n* (%)				0.171
None	149 (39.7)	20 (30.8)	129 (41.6)	
Continuous infusion	39 (10.4)	11 (16.9)	28 (9.0)	
Intermittent infusion	9 (2.4)	2 (3.1)	7 (2.3)	
Oral medication	178 (47.5)	32 (49.2)	146 (27.1)	

R-DWILs, remote diffusion-weighted imaging lesions; ICH, intracerebral hemorrhage; IS/TIA, ischemic stroke or transient ischemic attack; AF, atrial fibrillation; CHD, coronary heart disease; MRI, magnetic resonance imaging; NIHSS, National Institutes of Health Stroke Scale; WBC, white blood cell; TC, total cholesterol; LDL-C, low-density lipoprotein cholesterol; FBG, fasting blood glucose; CRP, C-reactive protein; WMH, white matter hyperintensity; IQR, interquartile range; SD, standard deviation; N, number.

**Figure 2:**
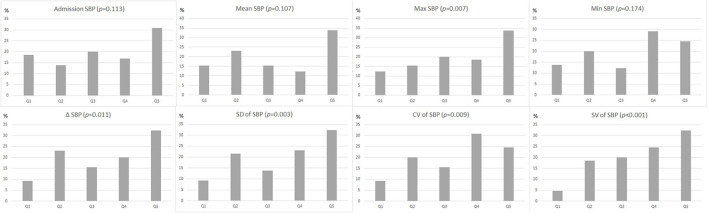
Proportion (as a percentage) of patients with R-DWILs according to quintiles (Q1: lowest quintile group, Q5: highest quintile group) of each blood pressure variability parameter. The *p*-values are for the χ^2^ test for linear trend.

In univariate analysis, patients with R-DWILs had greater SBP variability compared with those without, while evaluated by 3 indices of SD, CV, and SV respectively (Table 2). Other SBP variables associated with R-DWILs included mean SBP, max SBP, and ΔSBP. After these SBP variables were categorized into quintiles, the 3 BPV variables were all associated in a graded fashion with R-DWILs (*p*-values ranging from <0.01 to <0.001), as well as max (*p* = 0.007) andΔSBP (*p* = 0.011), while mean, minimal SBP or SBP on admission were not associated (Figure 2).

**Table 2:** Comparison of SBP profiles between patients with and without R-DWILs.

	**R-DWILs**			
	**All**	**No**	**Yes**	***P-*value**
*N* (%)	375 (100)	310 (82.7)	65 (17.3)	
Admission SBP, median (IQR)	162 (34)	160 (32)	166 (41)	0.066
Mean SBP, mean (SD)	144.2 (14.4)	143.4 (14.2)	147.8 (15.0)	*0.024*
Max SBP, mean (SD)	171.5 (22.5)	169.9 (21.7)	179.6 (24.9)	*0.001*
Min SBP, mean (SD)	122.4 (14.2)	122.0 (14.1)	124.4 (14.6)	0.226
ΔSBP, median (IQR)	46 (25)	46 (22)	50 (36)	*0.008*
SD SBP, median (IQR)	13.6 (6.9)	13.3 (6.4)	15.6 (7.7)	*0.002*
CV SBP, median (IQR)	9.5 (4.4)	9.4 (4.1)	10.9 (4.3)	*0.005*
SV SBP, median (IQR)	15.2 (6.9)	14.6 (7.0)	17.3 (6.3)	*<0.001*

R-DWILs, remote diffusion-weighted imaging lesions; SBP, systolic blood pressure; SD, standard deviation; CV, coefficient of variation; SV, successive variation; IQR, interquartile range; N, number. The italic values mean *p* < 0.05.

In multivariable logistic regression analysis, parameters of BPV were independently associated with R-DWILs, and the results remain consistent after adjusting with mean SBP (Table 3). SD, CV, and SV values in the highest quintile, showed 3- to 8-fold increased risk of R-DWILs, compared with the lowest quintile. ΔSBP also reached a significant difference in model 1 (*p* = 0.019), and marginally significant difference in model 2 (*p* = 0.050). Max SBP achieved significant difference in model 1 (*p* = 0.024), while failed to reveal significance in model 2 (*p* = 0.122). In contrast, mean SBP, admission SBP, min SBP, was not consistently associated with R-DWILs. Besides, we also applied a sensitivity analysis exploring the associations between SBP variability and R-DWILs number, and similar results were found (Supplementary Tables S1, S2).

**Table 3:** Effects of 1 quintile increment in BPV parameters and R-DWILs.

	**OR (95%CI)**					
	**Q1**	**Q2**	**Q3**	**Q4**	**Q5**	***P*-value**
**Model 1**						
Mean SBP	1	1.152 (0.443–2.997)	0.808 (0.291–2.242)	0.737 (0.256–2.118)	1.898 (0.752–4.790)	0.291
Admission SBP	1	0.728 (0.266–1.995)	0.804 (0.309–2.095)	0.853 (0.318–2.285)	1.920 (0.780–4.728)	0.126
Max SBP	1	1.275 (0.444–3.662)	1.775 (0.624–5.049)	1.304 (0.468–3.631)	3.361 (1.247–9.060)	*0.024*
Min SBP	1	1.725 (0.627–4.743)	0.610 (0.200–1.866)	1.892 (0.736–4.864)	1.609 (0.610–4.247)	0.309
ΔSBP	1	2.226 (0.748–6.624)	1.697 (0.550–5.238)	1.958 (0.659–5.814)	3.998 (1.407–11.359)	*0.019*
SD SBP	1	2.196 (0.738–6.537)	1.430 (0.460–4.444)	2.915 (0.996–8.534)	4.547 (1.588–13.017)	*0.004*
CV SBP	1	1.921 (0.641–5.760)	1.824 (0.588–5.659)	4.299 (1.509–12.250)	3.260 (1.113–9.549)	*0.007*
SV SBP	1	4.179 (1.058–16.514)	4.911 (1.262–19.117)	5.241 (1.388–19.791)	9.296 (2.477–34.896)	*0.001*
**Model 2**						
Mean SBP	–	–	–	–	–	–
Admission SBP	1	0.639 (0.226–1.807)	0.604 (0.201–1.819)	0.642 (0.209–1.976)	1.263 (0.380–4.195)	0.564
Max SBP	1	1.254 (0.405–3.884)	1.729 (0.504–5.930)	1.258 (0.327–4.840)	3.220 (0.760–13.649)	0.122
Min SBP	1	1.290 (0.440–3.777)	0.382 (0.107–1.370)	1.060 (0.322–3.485)	0.689 (0.162–2.924)	0.669
ΔSBP	1	2.126 (0.713–6.341)	1.528 (0.487–4.796)	1.764 (0.585–5.321)	3.457 (1.180–10.124)	0.050
SD SBP	1	2.130 (0.713–6.362)	1.230 (0.386–3.921)	2.557 (0.857–7.626)	3.977 (1.362–11.608)	*0.011*
CV SBP	1	1.737 (0.572–5.274)	1.874 (0.605–5.810)	3.920 (1.361–11.292)	3.244 (1.104–9.536)	*0.007*
SV SBP	1	3.951 (0.995–15.684)	4.544 (1.155–17.870)	4.630 (1.191–18.007)	8.411 (2.202–32.136)	*0.002*

Q1 means the lowest quintile group and Q5 means the highest quintile group of each SBP variable. Model 1 was adjusted for age, sex, initial National Institutes of Health Stroke Scale, fasting blood glucose, time to magnetic resonance imaging, hematoma volume, and high-grade white matter hyperintensity. Model 2 was adjusted for all variables in model 1 plus mean blood pressure. Abbreviations: R-DWILs, remote diffusion-weighted imaging lesions; BPV, blood pressure variability. SBP, systolic blood pressure; SD, standard deviation; CV, coefficient of variation; SV, successive variation; OR, odds ratio; CI, confidence interval. The italic values mean *p* < 0.05.

At 3 months, mRS assessments were completed on 335 patients (89.3%), and unfavorable outcomes (mRS 4–6) occurred in 84 patients (25.1%). In univariate analysis, BPV parameters were not associated with unfavorable outcomes at 3 months, although there was a tendency for increased SV SBP in the worse functional outcome group (*p* = 0.057, Supplementary Table S3).

In addition, parameters of DBP variability were not associated with R-DWILs in univariate analysis (Supplementary Table S4).

## Discussion

This study found that in the acute phase of ICH, BPV within the first 24 h after admission was strongly associated with R-DWILs independently. Furthermore, BPV was a more consistent and sensitive parameter related to R-DWILs than other classic measurements of BP magnitude, such as SBP on admission, maximum SBP, minimum SBP, and max SBP minus min SBP. BPV values located in the highest quintile showed an increased 3- to 8-fold risk of R-DWILs occurrence compared with the lowest quintile BPV values.

Blood pressure has been considered as an important therapeutic target in acute ICH since it is frequently elevated especially in the acute phase and higher values are associated with an increased risk of early deterioration, hematoma growth, and worse functional outcome (16, 17). However, highly rigorous BP reduction has not shown definite benefits in reducing mortality or severe disability in randomized trials (3, 4). It is argued that aggressive BP lowering may also increase the possibility of cerebral hypoperfusion, especially in the case of impaired cerebral autoregulation, and thus induce cerebral ischemia, which outweighs the benefit of reduction in hematoma growth (5). Prior studies have yielded conflicting results on the association between BP lowering and remote DWI lesions (2, 6–10). However, these analyses were based on only a few measurements of blood pressure, with the varied time intervals between ICH onset and image acquisition time. We examined various parameters of BP variability, including SV, a parameter that reflects the time sequence of measurements and may represent more physiologically relevant variation (18). In this context, our study provided more convincing evidence that DWI lesions are most likely associated with SBP variability instead of absolute decrease in SBP.

The association of SBP variability and R-DWILs indicated that fluctuation of SBP in the early stage of ICH may contribute to the formation of R-DWILs, which may have important implications for acute BP management in the setting of ICH. This hypothesis is supported by the fact that DWI lesions were noted more frequently in the acute stage of ICH compared with the non-acute stage (1), a period when BP change is the most dramatic. It is widely accepted that SBP variability in the early hours after ICH is closely related to death or early neurological deterioration or poor functional outcome within 90 days (19–21). Recently, a combined analysis of Intensive Blood Pressure Reduction in Acute Cerebral Hemorrhage 2 (INTERACT2) trial and the Antihypertensive Treatment of Acute Cerebral Hemorrhage II (ATACH-II) trial also revealed that a great change in blood pressure within the first hour was particularly associated with worse outcomes, and may be harmful (12). In these analyses, the authors suggested that poor prognosis may be caused by the promotion of hematoma enlargement and perihematomal edema due to significant BPV. Based on the results of our study, it is also possible that the association between BPV and poor prognosis may be partly due to the greater incidence of R-DWILs in these populations of greater BP fluctuation. The lack of association between BPV and unfavorable outcomes in our study may be due to the fact that we largely focused on BPV in the later time window, 24 h window after onset, when BP tends to be stable. Our findings are of particular interest since a growing body of evidence has shown that R-DWILs are not uncommon after ICH, and can result in a worse outcome, whereas the mechanism and etiology of these lesions remain unclear (2). However, our findings do not necessarily imply a direct causal relationship of BPV in determining R-DWILs. It is still possible that patients with R-DWILs may be more likely to experience BP variations.

The association between a max drop of SBP and R-DWILs in our study aligns with the findings of Kidwell et al.' s study enrolling 600 patients with primary ICH from the Ethnic/Racial Variations of Intracerebral Hemorrhage (ERICH) cohort, in which greater changes in mean arterial pressure (MAP) prior to the MRI were significantly associated with the presence of DWI lesions (7). Another study conducted by Buletko et al. also demonstrated that, after institutional protocol change in SBP target from <160 to <140 mmHg for acute ICH, there was an increased rate of cerebral ischemia in patients with SBP target <140 mm Hg (6). Conversely, in a more recent pooled analysis of 3 randomized clinical trials (Minimally Invasive Surgery Plus Alteplase for Intracerebral Hemorrhage Evacuation phase 3 [MISTIE III] trial, ATACH-II trial, and Intracerebral Hemorrhage Deferoxamine [i-DEF] phase 2 trial) and 1 multicenter prospective study (ERICH), which was conducted by Murthy et al., SBP reduction was not associated with DWI lesions (2). However, data on BP was mostly available only at 2 time points, which surely cannot reflect real BP control status during the acute phase of ICH. Besides, we found that median SBP changes over 24 h, were similar to the drop seen in patients in Kidwell et al.' s (7) and Buletko et al.' s study (6), while SBP drop in Murthy et al.' s study is more stable (2), which may have resulted in the negative association between BP reduction and R-DWILs in Murthy et al.' s study. The lack of association between min SBP and R-DWILs also indicates that it is the procedure of BP reduction that matters, rather than the particular SBP target. Further studies may be warranted to dogmatically determine whether or to what extent BP reduction is needed.

We also found that max SBP achieved a significant difference in model 1, but did not remain significant after adjusting for mean SBP in model 2. Besides, we failed to find an association between admission SBP and R-DWILs. In prior studies, several studies have reported an association with admission SBP (2, 7, 8), whereas other studies have reported null results (10, 14). It is assumed that higher SBP may precipitate cerebral vasospasm, which leads to small DWI lesions (7). Given this assumption, max SBP during the initial hours of ICH may be more closely linked with R-DWILs compared with admission SBP. The reason why the association between max SBP and R-DWILs was not shown in model 2 may partly be due to the small sample size, and this requires confirmation in larger prospective studies.

It is noteworthy in the present study that the variability parameters for SBP were more closely related to R-DWILs than those for DBP. Previous studies about BP variability and ICH outcome have also reported that SBP variability might be more closely correlated with ICH outcome than either mean DBP or DBP variability (21, 22), although the reasons are still unrevealed.

This study has several limitations. First, our cohort may have a selection bias toward patients with milder hemorrhages due to MRI image requirements. Second, we did not differentiate varieties of BP-lowering medications, resulting in the inability to assess the effects of specific antihypertensive agents. Third, we did not assess the impact of BPV in the hyperacute period of ICH due to the limitation of patients, during which hemodynamic changes are most prominent. Furthermore, given the small size of R-DWILs, 6 mm slice thickness with 0.5 mm gap to acquire DWI sequences could cause a large measuring error, as well as T2 shine-through calculated based on b0.

In conclusion, the results of our study revealed that SBP variability during the first 24 h after ICH ictus was independently associated with remote DWI lesions. This finding implies an effort to maintain BP stably instead of rigorously during the acute stage of ICH. Further studies with longer and more comprehensive BP surveillance are required to confirm the influence of blood pressure on not only R-DWILs but also hematoma growth and neurological outcome after ICH.

## Data availability statement

The original contributions presented in the study are included in the article/Supplementary material, further inquiries can be directed to the corresponding author/s.

## Ethics statement

The studies involving human participants were reviewed and approved by the Institutional Human Research Ethics Committee of the Second Affiliated Hospital of Zhejiang University. The patients/participants provided their written informed consent to participate in this study. Written informed consent was obtained from the individual(s) for the publication of any potentially identifiable images or data included in this article.

## Author contributions

XX and SP interpreted the data and drafted the manuscript. YZ performed the statistical analyses. JL collected the data. LT and FG read the images, revised the manuscript, and supervised the whole framework. All authors contributed to the article and approved the submitted version.

## Funding

This work was supported by the National Natural Science Foundation of China (NSFC) (81471168), the National Natural Science Foundation of China (NSFC) (81971155), Science and Technology Action Plan for Major Diseases Prevention and Control in China (2017ZX-01S-006S3), the Science and Technology Department of Zhejiang Province (2022KY174), and the Basic research Fund of Zhejiang University (226-2022-00027).

## Conflict of interest

The authors declare that the research was conducted in the absence of any commercial or financial relationships that could be construed as a potential conflict of interest.

## Publisher's note

All claims expressed in this article are solely those of the authors and do not necessarily represent those of their affiliated organizations, or those of the publisher, the editors and the reviewers. Any product that may be evaluated in this article, or claim that may be made by its manufacturer, is not guaranteed or endorsed by the publisher.
