# Supplementary material for: Remote diffusion-weighted imaging lesions and blood pressure variability in primary intracerebral hemorrhage
Source: Front Neurol. 2022 Sep 20;13:950056. doi: 10.3389/fneur.2022.950056 (PMC9530136; doi:10.3389/fneur.2022.950056)
Supplement: Supplementary file 1 [file Data_Sheet_1.docx]

Supplementary Material

## Supplementary Tables

**Supplementary Table 1**. Spearman’s correlation analysis between SBP profiles and R-DWILs number

|  | R-DWILs number | |
| --- | --- | --- |
|  | *r* | *p* |
| Admission SBP | 0.097 | 0.059 |
| Mean SBP | 0.097 | 0.062 |
| Max SBP | 0.145 | *0.005* |
| Min SBP | 0.054 | 0.299 |
| ΔSBP | 0.135 | *0.009* |
| SD SBP | 0.162 | *0.002* |
| CV SBP | 0.144 | *0.005* |
| SV SBP | 0.208 | *<0.001* |

R-DWILs, remote diffusion-weighted imaging lesions; SBP, systolic blood pressure; SD, standard deviation; CV, coefficient of variation; SV, successive variation.

**Supplementary Table 2**. Multiple linear regression analysis between BPV parameters and R-DWILs number

|  | Model 1 | | Model 2 | | |
| --- | --- | --- | --- | --- | --- |
|  | β | *P* Value | | β | *P* Value |
| Mean SBP | 0.040 | 0.469 | | - | - |
| Admission SBP, median | 0.103 | 0.057 | | 0.135 | 0.059 |
| Max SBP, mean | 0.123 | *0.024* | | 0.229 | *0.007* |
| Min SBP, mean | 0.014 | 0.797 | | -0.063 | 0.521 |
| ΔSBP, median | 0.125 | *0.018* | | 0.123 | *0.025* |
| SD SBP, median | 0.134 | *0.012* | | 0.133 | *0.016* |
| CV SBP, median | 0.124 | *0.017* | | 0.125 | *0.017* |
| SV SBP, median | 0.155 | *0.004* | | 0.154 | *0.005* |

Model 1 was adjusted for age, sex, initial National Institutes of Health Stroke Scale, fasting blood glucose, time to magnetic resonance imaging, hematoma volume, and high-grade white matter hyperintensity. Model 2 was adjusted for all variables in model 1 plus mean blood pressure. Abbreviations: R-DWILs, remote diffusion-weighted imaging lesions; BPV, blood pressure variability. SBP, systolic blood pressure; SD, standard deviation; CV, coefficient of variation; SV, successive variation.

**Supplementary Table 3.** Univariate analysis of BPV parameters associated with 3-month mRS outcomes

|  | 3-month mRS outcomes | | | |
| --- | --- | --- | --- | --- |
|  | All | Good | Poor | P Value |
| N (%) | 335(100) | 84(25.1) | 251(74.9) |  |
| Admission SBP, median (IQR) | 162(34) | 159(30) | 165(34) | 0.433 |
| Mean SBP, mean (SD) | 144.1(14.4) | 145.1(15.9) | 144.4(13.7) | 0.694 |
| Max SBP, mean (SD) | 171.5(22.5) | 171.6(22.6) | 172.2(22.7) | 0.840 |
| Min SBP, mean (SD) | 122.4.0(14.2) | 122.7(15.5) | 123.0(13.8) | 0.893 |
| ΔSBP, median (IQR) | 46.0(25) | 45.5(22) | 46.0(25) | 0.936 |
| SD SBP, median (IQR) | 13.6(6.9) | 13.4(6.8) | 13.4(6.9) | 0.884 |
| CV SBP, median (IQR) | 9.5(4.4) | 9.4(3.8) | 9.4(4.4) | 0.985 |
| SV SBP, median (IQR) | 15.2(6.9) | 15.8(7.2) | 14.9(7.1) | 0.057 |

mRS, modified rankin scale; SBP, systolic blood pressure; SD, standard deviation; CV, coefficient of variation; SV, successive variation; IQR, interquartile range; N, number.

**Supplementary Table 4.** Comparison of DBP profiles between patients with and without R-DWILs

|  | R-DWILs | | | |
| --- | --- | --- | --- | --- |
|  | All | No | Yes | *P* Value |
| N (%) | 375(100) | 310(82.7) | 65(17.3) |  |
| Admission DBP, median (IQR) | 91(20) | 90(19) | 95(24) | 0.159 |
| Mean DBP, mean (IQR) | 83(15) | 83(15) | 84(16) | 0.579 |
| Max DBP, mean (IQR) | 99(19) | 99(19) | 100(21) | 0.218 |
| Min DBP, mean (SD) | 67.3(10.8) | 67.2(11.0) | 67.7(10.1) | 0.749 |
| ΔDBP, median (IQR) | 31(16) | 31(16) | 32(17) | 0.190 |
| SD DBP, median (IQR) | 9.0(4.3) | 9.0(4.4) | 9.3(4.1) | 0.361 |
| CV DBP, median (IQR) | 10.9(5.0) | 11.0(5.2) | 11.5(4.8) | 0.523 |
| SV DBP, median (IQR) | 11.2(5.0) | 10.8(5.2) | 11.2(4.3) | 0.386 |

R-DWILs, remote diffusion-weighted imaging lesions; DBP, diastolic blood pressure; SD, standard deviation; CV, coefficient of variation; SV, successive variation; IQR, interquartile range; N, number.

## Supplementary Figures


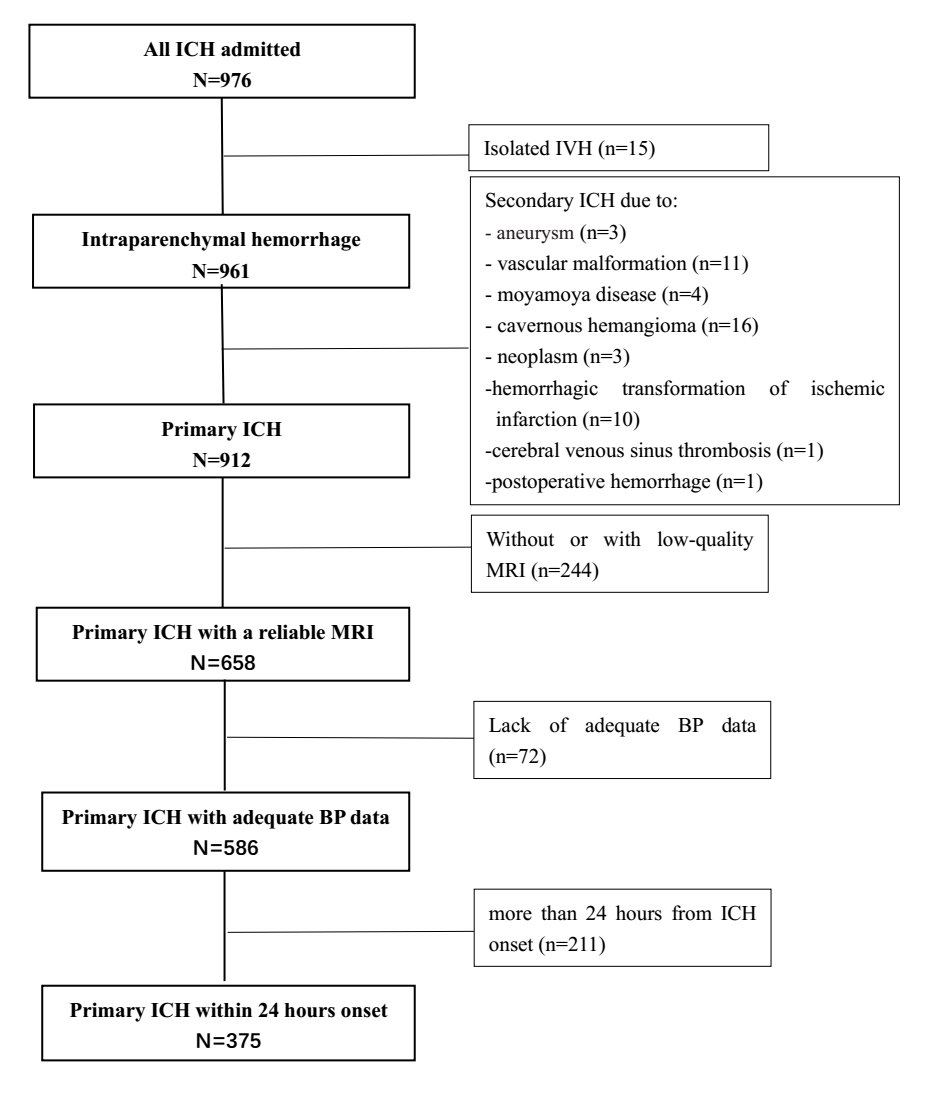


**Supplementary Figure 1.** Cohort ﬂowchart. Abbreviations: ICH, intracerebral hemorrhage; IVH, intraventricular hemorrhage; MRI, magnetic resonance imaging; BP, blood pressure; N, number.
